# UHRF1 regulation of the Keap1–Nrf2 pathway in pancreatic cancer contributes to oncogenesis

**DOI:** 10.1002/path.4665

**Published:** 2015-11-30

**Authors:** Wafa Abu‐Alainin, Thompson Gana, Triantafillos Liloglou, Adedamola Olayanju, Lawrence N Barrera, Robert Ferguson, Fiona Campbell, Timothy Andrews, Christopher Goldring, Neil Kitteringham, Brian K Park, Taoufik Nedjadi, Michael C Schmid, Joseph R Slupsky, William Greenhalf, John P Neoptolemos, Eithne Costello

**Affiliations:** ^1^ Department of Molecular and Clinical Cancer MedicineUniversity of LiverpoolUK; ^2^ Roy Castle Lung Cancer Research Programme, Department of Molecular and Clinical Cancer MedicineUniversity of LiverpoolUK; ^3^ Department of Pharmacology and TherapeuticsUniversity of LiverpoolUK; ^4^ Department of PathologyRoyal Liverpool University HospitalUK

**Keywords:** UHRF1, DNA methylation, Keap1, Nrf2, pancreatic cancer

## Abstract

The cellular defence protein Nrf2 is a mediator of oncogenesis in pancreatic ductal adenocarcinoma (PDAC) and other cancers. However, the control of Nrf2 expression and activity in cancer is not fully understood. We previously reported the absence of Keap1, a pivotal regulator of Nrf2, in ∼70% of PDAC cases. Here we describe a novel mechanism whereby the epigenetic regulator UHRF1 suppresses Keap1 protein levels. UHRF1 expression was observed in 20% (5 of 25) of benign pancreatic ducts compared to 86% (114 of 132) of pancreatic tumours, and an inverse relationship between UHRF1 and Keap1 levels in PDAC tumours (n = 124) was apparent (p = 0.002). We also provide evidence that UHRF1‐mediated regulation of the Nrf2 pathway contributes to the aggressive behaviour of PDAC. Depletion of UHRF1 from PDAC cells decreased growth and enhanced apoptosis and cell cycle arrest. UHRF1 depletion also led to reduced levels of Nrf2‐regulated downstream proteins and was accompanied by heightened oxidative stress, in the form of lower glutathione levels and increased reactive oxygen species. Concomitant depletion of Keap1 and UHRF1 restored Nrf2 levels and reversed cell cycle arrest and the increase in reactive oxygen species. Mechanistically, depletion of UHRF1 reduced global and tumour suppressor promoter methylation in pancreatic cancer cell lines, and KEAP1 gene promoter methylation was reduced in one of three cell lines examined. Thus, methylation of the KEAP1 gene promoter may contribute to the suppression of Keap1 protein levels by UHRF1, although our data suggest that additional mechanisms need to be explored. Finally, we demonstrate that K‐Ras drives UHRF1 expression, establishing a novel link between this oncogene and Nrf2‐mediated cellular protection. Since UHRF1 over‐expression occurs in other cancers, its ability to regulate the Keap1–Nrf2 pathway may be critically important to the malignant behaviour of these cancers. © 2015 The Authors. *Journal of Pathology* published by John Wiley & Sons Ltd on behalf of Pathological Society of Great Britain and Ireland.

## Introduction

Pancreatic cancer has a dismally low overall 5 year survival rate 1, 2. This disease is characterized by genetic and epigenetic changes within malignant cells that play fundamental roles in its development and progression 3. DNA methylation is the best‐studied epigenetic mechanism influencing pancreatic cancer development through the inactivation of tumour suppressor gene promoters, such as *CDKN2A* (p16^INK4a^) 4 and others 5. Our previous work reported the absence of Kelch‐like ECH‐associated protein 1 (*Keap1*) expression in ∼70% of PDACs. The Keap1–nuclear factor erythroid 2‐related factor 2 (Nrf2) pathway protects normal cells from chemical or oxidative stress and is up‐regulated in pancreatic cancer 6, 7. Keap1 interacts with the transcription factor Nrf2, directing it for ubiquitylation and proteasomal degradation. Disruption of this interaction results in stabilization and nuclear accumulation of Nrf2, where it interacts with antioxidant response elements (AREs), promoting the transcription of genes encoding antioxidant proteins, detoxification enzymes and xenobiotic transporters 8. The protection from oxidative stress and chemotherapeutic agents thus afforded by Nrf2 may facilitate cancer progression 8. Disruption between Nrf2 and Keap1 may occur through different mechanisms, which are still not fully established 9. However, an emerging mechanism is methylation of the *KEAP1* gene promoter, seen in a variety of common cancers 10, 11, 12, 13, 14.

DNA methyltransferase 1 (Dnmt1) is largely responsible for maintaining DNA methylation patterns from the parent strand of DNA to the newly synthesized daughter strand 15. Ubiquitin‐like containing PHD and RING finger domains 1(UHRF1; also called ICBP90 in humans and Np95 in mice) contributes to the maintenance of DNA methylation by recruiting Dnmt1 to its hemimethylated DNA substrate 16. UHRF1 is a multi‐domain protein important for cell growth 17 and is over‐expressed in breast 18, 19, bladder 20, 21, colorectal 22, 23, lung 24, 25 prostate 26 and pancreatic cancers 27.

Here we report an important novel function for UHRF1 in controlling Keap1 protein levels and, consequently, the activity of Nrf2. The expression of UHRF1 in pancreatic cancer cells stimulates growth and protects from stress through increasing Nrf2 activity. Since UHRF1 is over‐expressed in several other cancers, its ability to regulate Keap1–Nrf2 may be important in their pathogenesis.

## Materials and methods

### Cell culture

Human PDAC cell lines, MiaPaca‐2, Panc‐1, CFpac‐1 (American Type Culture Collection, ATCC) and SUIT‐2 28, were cultured as described previously 7. Primary mouse pancreatic cancer cell lines were isolated from tumours arising in K‐Ras^LSL‐G12D/+^, p53^R172 h/+^ and Pdx1–Cre mice (KPC) 29. Low passage (<10) KPC cells were used.

### Small interference RNA (siRNA)‐mediated knockdown of UHRF1 and Keap1

Typically, 2 × 10^5^ cells were seeded in six‐well plates (Corning B.V. Life Sciences, Amsterdam, The Netherlands) and were transfected at 40% confluency with 30 nm siRNA, using Lipofectamine 2000 (Life Technologies) in antibiotic‐free medium, according to the manufacturer's instructions, and harvested at 72 h or indicated times. For details and primer sequences, see supplementary material, Supplementary materials and methods.

### Western blotting

Whole‐cell lysates were prepared, using buffer containing 100 mm Tris–HCl, pH 6.8, 2% sodium dodecyl sulphate and protease inhibitors (Roche). Western blotting was performed as described previously 30. For details of the antibodies used, see supplementary material, Supplementary materials and methods.

### Luciferase assay

One thousand cells/well were seeded into 96‐well plates and transfected with control, UHRF1 and Nrf2 siRNA (10 nm). After 48 h, the cells were transfected with a pGL4 luciferase reporter plasmid (Promega, Madison, WI, USA) containing eight antioxidant response elements (AREs) in a final volume of 100 µl/well and luciferase assays undertaken 24 h later.

### Proliferation and apoptosis assays

Cell proliferation was measured using the MTS EZ4U Kit (Fa. Biomedica, Vienna, Austria) 31. For analysis of apoptosis, the activity of caspase 3/7 was measured using a Caspase‐Glo 3/7 Assay Kit (Promega) following treatment of selected wells with the general caspase inhibitor ZV AD (30 µm).

### Immunohistochemistry and immunocytochemistry

A tissue micro‐array (TMA) containing PDAC tissue, obtained with informed consent and ethical approval (North West 1 Research Ethics Committee; Ref. No. 11/NW/0083) from 132 patients treated at the Royal Liverpool University Hospital, UK, was stained using two UHRF1 antibodies (ab57083, Abcam; sc‐136264, Santa Cruz Biotechnology) and anti‐Keap1 (sc‐15246, Santa Cruz Biotechnology) antibodies at dilutions of 1:200 and 1:100, respectively 32, 33. Scoring was performed by two pancreatic specialist histopathologists (FC and TA). For UHRF1, the intensity of nuclear staining was recorded (graded 0 = negative, 1 = weak, 2 = moderate and 3 = strong). Keap1 cytoplasmic or membranous staining was scored as either positive or negative 7. Immunocytochemistry (ICC) for UHRF1 was performed as described previously 32.

### DNA methylation analysis

Pyrosequencing assays (Qiagen) were undertaken to measure methylation levels of *LINE‐1*, *RASSF1*, *CDKN2A* and *KEAP1* promoters, as described previously 25. Primer sequences for each promoter region analysed are shown in Figure S1 (see supplementary material). The promoter methylation index of all examined CpGs was calculated as the mean value of *mC*/(*mC* + *C*), where *C* is unmethylated cytosine and *mC* is 5′‐methyl cytosine. Experiments were repeated and differences between control and UHRF1‐depleted conditions examined using Wilcoxon's test.

### Double thymidine block and flow cytometry

Cells grown to 40–50% confluence were treated with 2 mm thymidine (Sigma‐Aldrich) for 19 h, washed three times, the medium added and incubated for a further 9 h. Then 2 mm thymidine was added and the cells incubated for 16 h. The cells were washed with PBST, thymidine‐free medium added and cells collected at 0, 2, 4, 7, 8, 9 and 11 h for lysate preparation or for flow cytometry.

### Analysis of oxidative stress

ROS generation was measured by incubating cells with 2′,7′‐dichlorodihydrofluorescein diacetate (H2DCFDA; Life Technologies, Paisley, UK) for 30 min, followed by flow cytometry. Total glutathione (GSH) content of cells (5 × 10^5^) in six‐well plates was quantified using the 5,5′‐dithiobis‐2‐nitrobenzoic acid–GSH reductase recycling method 34. Sample GSH concentrations were calculated via reference to a standard curve and normalized to total protein.

### RNA extraction and quantitative real‐time PCR (qRT–PCR)

RNA was extracted (RNeasy Kit, Qiagen) and cDNA synthesized [Promega Improm II Reverse Transcriptase (RT) Kit]. Quantitative real‐time PCR (qRT–PCR) was undertaken for *KEAP1* and *GAPDH* (for primers, see supplementary material, Supplementary materials and methods). Data were analysed using ABI PRISM 7000 SDS software, SYBR green template mode. The assay was performed three times in duplicate, with *GAPDH* used for normalization.

### Data analysis

Statistical analyses were performed using StatView v. 5.0.1. (SAS Institute, Cary, NC, USA). To obtain associations between UHRF1 expression and clinical parameters, data were cross‐tabulated and Fisher's two‐sided exact test or Mann–Whitney U‐test performed. Survival analysis was performed using the Kaplan–Meier test. Spearman's rank correlation was used to correlate agreement of nuclear UHRF1 staining percentage between Abcam antibody and Santa Cruz antibodies. Results were considered significant at *p* < 0.05.

## Results

### UHRF1 over‐expression in pancreatic cancer is associated with larger tumour size

**Figure 1:**
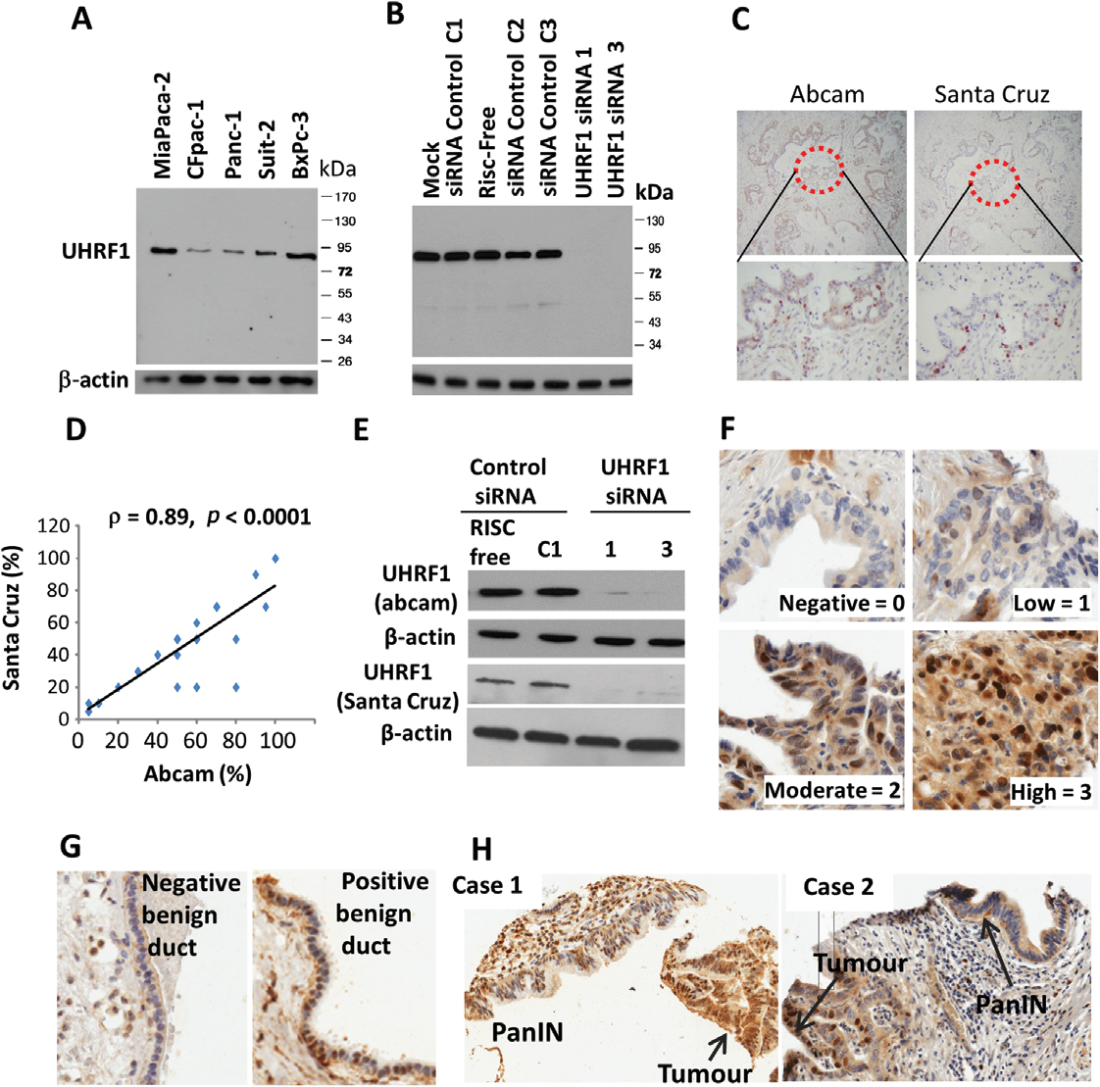
UHRF1 is over‐expressed in pancreatic cancer. (A) Western blot of UHRF1 in a panel of pancreatic cancer cell lines. (B) UHRF1 expression in MiaPaca‐2 cells following transfection with control siRNAs (C1, C2, C3), Risc‐Free or UHRF1‐targeting siRNAs 1 and 3. (C) Immunohistochemical staining of UHRF1 with Abcam and Santa Cruz UHRF1 antibodies. (D) Correlation of UHRF1‐positive nuclei stained with Abcam or Santa Cruz antibodies in 32 cases. (E) Western blot (MiaPaca‐2 cells) for UHRF1, with Abcam and Santa Cruz antibodies, following the indicated treatments. (F) UHRF1 levels in pancreatic cancer tissue, scored 0–3 according to the intensity of nuclear UHRF1 expression. UHRF1 in (G) benign ducts and (H) matched PanIN and tumour samples, showing a positively stained PanIN (case 1) and a PanIN lacking UHRF1 (case 2)

Western blotting revealed variable levels of UHRF1 in pancreatic cancer cells (Figure 1A). The single predominant 90 kDa band for UHRF1 was lost following treatment with UHRF1‐targeting siRNA, but not after control treatments (Figure 1B). As part of the process of antibody validation for immunohistochemistry, two independent antibodies (from Abcam and Santa Cruz) were evaluated in 32 pancreatic cancer cases (Figure 1C). Both antibodies detected nuclear staining, whilst only the Abcam antibody stained the cytoplasm. Our study therefore focused on nuclear UHRF1 expression. The percentages of positive nuclei detected by each antibody were highly correlated (ρ = 0.89, *p* < 0.001; Figure 1D) providing strong evidence of specificity for nuclear UHRF1. Moreover, western analysis gave similar results with both antibodies (Figure 1E).

Immunohistochemical analysis of a pancreatic cancer tissue micro‐array revealed nuclear staining in 114 of 132 tumours (86%). Staining was variable between samples (Figure 1F; 18 tumours scored 0 (negative), 36 scored 1 (weak), 48 scored 2 (moderate) and 30 scored 3 (strong). For 25 cases, benign ducts were evaluable; 20 (80%) lacked nuclear UHRF1, while the remaining five were positive for nuclear UHRF1 staining (Figure 1G). Of note, matched PanIN lesions were available for eight UHRF1‐positive tumours; PanIN lesions express UHRF1 at variable but generally lower levels than matched tumours (Figure 1H).

Nuclear UHRF1 levels were dichotomized into negative (score 0) or positive (score 1, 2 or 3). UHRF1 was not associated with age (< or > 60 years, Fisher's exact test, p = 0.35), gender (Fisher's exact test, p = 0.61), lymph node status (Fisher's exact test, p = 0.99), grade (poor, moderate, good; χ^2^ = 4.8, p = 0.09;) or outcome (Kaplan–Meier log‐rank test, p = 0.98). However, positive expression of UHRF1 was associated with larger tumour size (>20 mm diameter, as measured in pathology; Fisher's exact test, p = 0.02). Of 114 tumours positive for UHRF1, 94 (82%) were > 20 mm diameter, compared to 55% (10 of 18) of cases lacking UHRF1. In summary, the majority of pancreatic tumours over‐express nuclear UHRF1 and its over‐expression is associated with larger tumours.

### Depletion of UHRF1 is associated with loss of global and tumour suppressor promoter methylation

**Figure 2:**
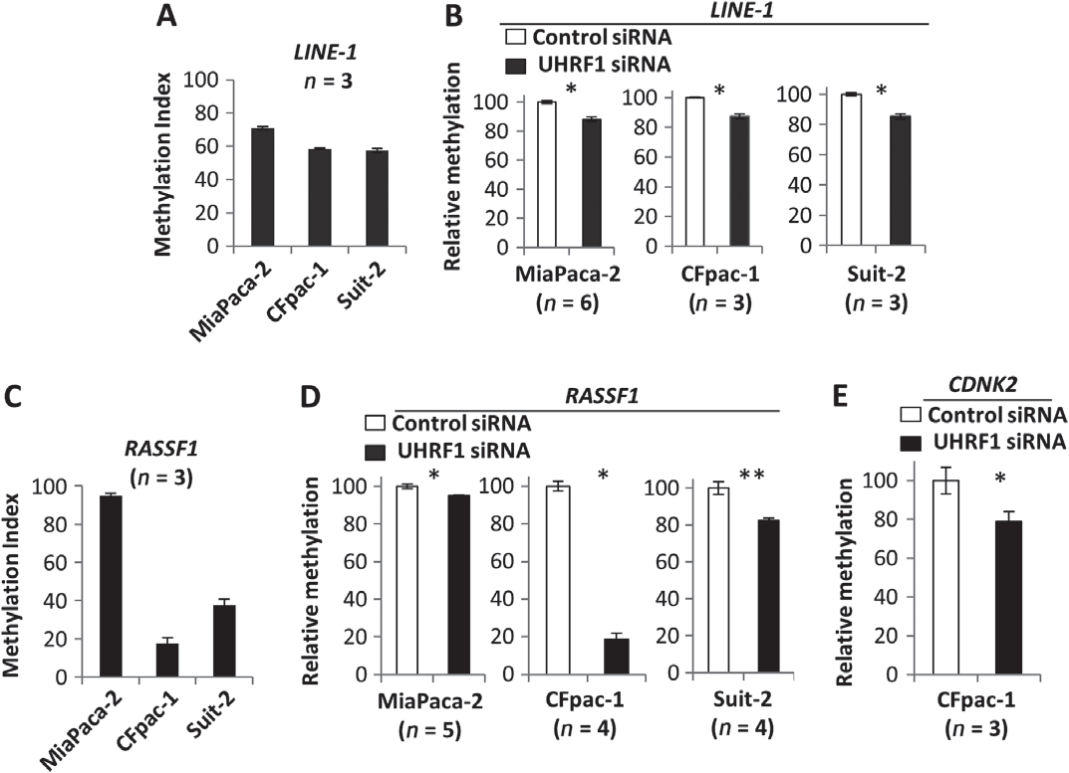
UHRF1 contributes to the maintenance of DNA methylation in pancreatic cancer cells. (A, C) Basal methylation index detected by triplicate pyrosequencing experiments of the LINE1 and RASSF1 promoters. (B, D, E) Mean of DNA methylation measurements of the (B) LINE1, (D) RASSF1 and (E) CDKN2A promoters following control‐ or UHRF1‐targeting siRNAs; *p < 0.01

We previously reported a lack of Keap1 expression in 70% of PDAC cases 7 and wished to ascertain whether the high levels of UHRF1 observed in PDAC could promote silencing of the KEAP1 promoter. However, since no data relating to the potential contribution of UHRF1 to the maintenance of DNA methylation in PDAC were available, we initially examined whether expression levels of this protein influenced either global (LINE‐1) methylation or tumour suppresser promoter (RASSF1, CDKN2A) methylation in PDAC. Depletion of UHRF1 reduced methylation levels of the LINE‐1 promoter in MiaPaca‐2, CFpac‐1 and Suit‐2 cells by 12%, 13% and 15%, respectively (Figure 2A, B). Similarly, UHRF1 depletion decreased promoter methylation of RASSF1 (Figure 2C, D; see also supplementary material, Figures S2, S3A) by 5%, 80% and 17%, respectively. When analysing methylation of the CDKN2A tumour suppressor gene, we used CFpac‐1 cells because this gene is fully deleted in MiaPaca‐2 and is mutated in Suit‐2 cells. The basal level of CDKN2A promoter methylation (93%) was reduced by 21% following UHRF1 knockdown (Figure 2E; see also supplementary material, Figure S3B).

### Depletion of UHRF1 is associated with loss of KEAP1 promoter methylation and deactivation of the Nrf2–Keap1 pathway

**Figure 3:**
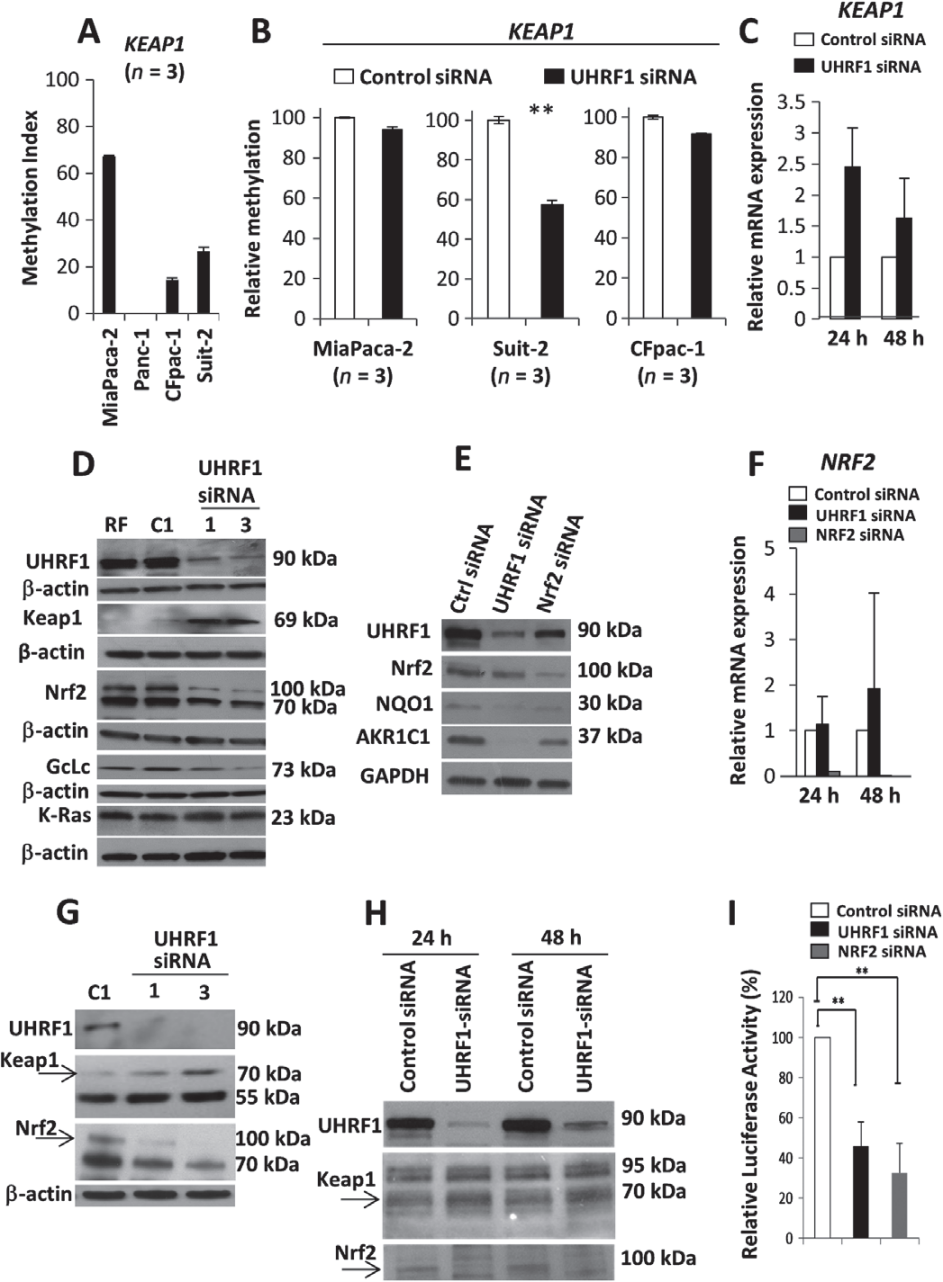
UHRF1 regulates the Nrf2–Keap1 pathway by maintaining KEAP1 promoter methylation. (A) Basal methylation index detected by triplicate pyrosequencing of KEAP1. (B) Mean of triplicate DNA methylation measurements of KEAP1 promoter in MiaPaca‐2, Suit2 and CFpac‐1 cells following control‐ or UHRF1‐targeting siRNAs. (C) RT–PCR for KEAP1 transcripts relative to GAPDH in control and UHRF1‐depleted Suit‐2 cells. (D, E) Western blot (Suit‐2) of the indicated proteins following UHRF1 or Nrf2 depletion. (F) RT–PCR for NRF2 transcripts relative to GAPDH in control and UHRF1‐ or Nrf2‐depleted Suit‐2 cells. (G, H) Gain in Keap1, down‐regulation of Nrf2 in MiaPaca‐2 and primary PDAC cells from a KPC mouse, respectively. (I) Relative luciferase activity from an 8× ARE‐reporter following UHRF1 or Nrf2 depletion (Suit‐2);**p < 0.01

We next turned our attention to the *KEAP1* promoter, asking whether the *KEAP1* gene promoter is silenced through methylation in pancreatic cancer cell lines. Basal levels of *KEAP1* gene promoter methylation were observed for MiaPaca‐2 (68%), Suit2 (27%) and CFpac‐1 (15%) cells, although no *KEAP1* promoter methylation was found in Panc‐1 cells (Figure 3A). Depletion of UHRF1 resulted in a loss of *KEAP1* promoter methylation in MiaPaca‐2, Suit2 and CFpac‐1 cells of 6%, 42% and 9%, respectively (Figure 3B), although this reached statistical significance in Suit‐2 cells only. We next sought to determine whether UHRF1 depletion was accompanied by enhanced *KEAP1* gene transcription. In Suit‐2 cells, *Keap1* mRNA increased at 24 h, declining at 48 and 72 h post‐depletion (Figure 3C). Moreover, UHRF1 depletion led to an increase in Keap1 protein levels at 24, 48 and 72 h (see supplementary material, Figure S4A), a concomitant loss of Nrf2 protein levels and loss in Nrf2 downstream proteins 35, 36, glutamate–cysteine ligase catalytic subunit (GcLc), aldo‐keto reductases 1C1 (AKR1C1) and NAD(P)H:quinone oxidoreductase 1 (NQO1) (Figure 3D, E). We could not establish a decrease in Nrf2 transcripts, although depletion of Nrf2 itself caused down‐regulation of Nrf2 transcripts (Figure 3F). Of note, K‐Ras, a potent oncogenic driver of pancreatic cancer, which lies upstream of Nrf2 6, was unaffected by UHRF1 depletion (Figure 3E) from Suit‐2 cells, which harbour oncogenic K‐Ras. UHRF1 depletion also led to altered Keap1 protein and Nrf2 protein levels in MiaPaca‐2 cells (Figure 3G), primary PDAC cells from KPC mice (Figure 3H) and CFpac‐1 (see supplementary material, Figure S4B). Finally, antioxidant response element (ARE)–luciferase activity was diminished in response to UHRF1 or Nrf2 depletion in Suit‐2 (Figure 3I) and MiaPaca‐2 (see supplementary material, Figure S4C) cells. Our data suggest that the Keap1–Nrf2 pathway is activated in pancreatic cancer cell lines through UHRF1‐mediated suppression of Keap1 protein levels, with Keap1 promoter methylation potentially contributing to this.

### UHRF1 suppression of Keap1 is required for protection from oxidative stress and optimal growth

**Figure 4:**
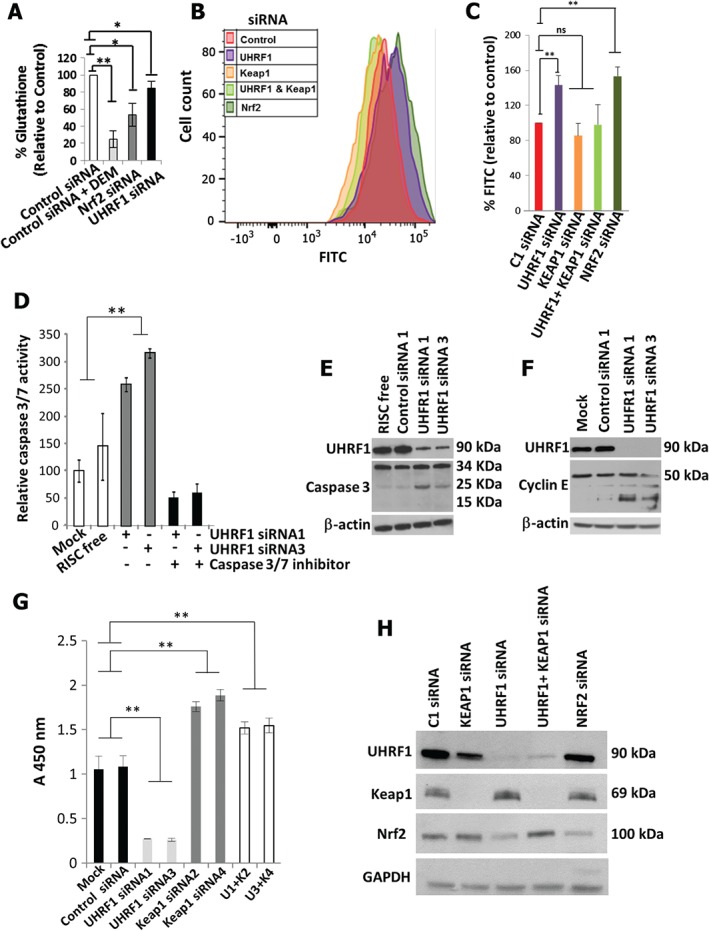
UHRF1 contributes to pancreatic cancer cell growth through activation of Nrf2. (A) GSH levels and (B, C) reactive oxygen species levels following the indicated treatments in MiaPaca‐2 cells (n = 3). (D) Caspase 3/7 activity in Suit‐2 cells following the indicated treatments (n = 3). (E, F) Western blot of cleaved caspase 3 and cleaved cyclin E following UHRF1 depletion from Suit‐2 cells (n = 3 independent experiments). (G) MTS assay of MiaPaca‐2 cells 72 h after control treatments or depletion of UHRF1, Keap1 or both; U1 + K2 = UHRF1 siRNA1 + Keap1 siRNA2; U3 + K4 = UHRF1 siRNA3 + Keap1 siRNA4. (H) Western analysis of MiaPaca‐2 cells following the indicated treatments; *p < 0.05; ** p < 0.01

As the intracellular redox state is predominantly regulated by Nrf2, we examined whether down‐regulation of the Keap1–Nrf2 pathway following UHRF1 depletion increases oxidative stress. Treatment of MiaPaca‐2 cells with the GSH depleter diethyl maleate (DEM) led to a significant decrease in total glutathione (GSH) levels, as did siRNA‐mediated depletion of Nrf2. UHRF1 depletion also resulted in a modest, although significant, decrease in GSH (Figure 4A). We also examined reactive oxygen species (ROS). Depletion of Nrf2 from MiaPaca‐2 cells caused an increase in ROS, as expected, while depletion of Keap1 did not (Figure 4B, 4C). Depletion of UHRF1 was also accompanied by increased ROS, an effect that was rescued by the simultaneous depletion of UHRF1 and Keap1. Similar results were observed in Suit‐2 cells (see supplementary material, Figure S4D). Thus, loss of UHRF1 is accompanied by an increase in cellular oxidative stress, suggesting that UHRF1, through the suppression of Keap1 expression, activates Nrf2, which protects pancreatic cancer cells from oxidative stress.

UHRF1 depletion from all pancreatic cancer cell lines examined was accompanied by diminished cell numbers, as evidenced by phase‐contrast microscopy, immunocytochemistry and MTS assay (see supplementary material, Figure S5). Diminished growth was accompanied by increased apoptosis, as revealed by elevated caspase 3/7 activity (Figure 4D) and increased caspase 3 and Cyclin E cleavage (Figure 4E, F). Since UHRF1 depletion is accompanied by elevated Keap1 levels, we sought to determine whether the decrease in cell growth induced by UHRF1 depletion could be abrogated by simultaneously depleting Keap1. Depletion of Keap1 alone led to a substantial increase in the growth of pancreatic cancer cells, whereas depletion of UHRF1 alone led to a reduction (Figure 4G). Modulation of growth kinetics corresponded, respectively, with an increase or decrease in Nrf2 protein expression, which, in the latter case, was reduced to a level similar to that observed in cells treated with Nrf2 siRNA (Figure 4H). Simultaneous depletion of both Keap1 and UHRF1 reversed the inhibitory effect of depleted UHRF1 alone on cell growth (Figure 4G) and also restored Nrf2 expression (Figure 4H). That Nrf2 is important for PDAC cell growth was established by our previous work showing that knockdown of Nrf2 dramatically inhibits PDAC cell proliferation 7. Taken together, these data indicate that UHRF1 promotes the growth of pancreatic cancer cells by reducing Keap1 levels and inducing activation of Nrf2.

### UHRF1 depletion induces a G_2_–M cell cycle block, which is abrogated by simultaneous depletion of Keap1

**Figure 5:**
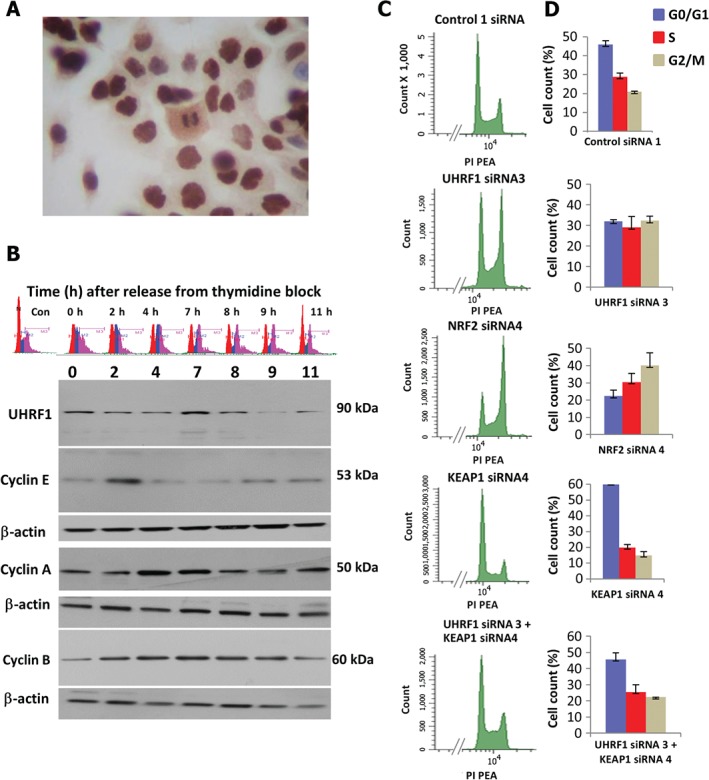
UHRF1 suppression of Keap1 is required for efficient transition of pancreatic cancer cells through G_2_–M. (A) ICC of CFpac‐1 cells, showing variable UHRF1 expression. (B) UHRF1, cyclin E, A and B western blotting of CFpac‐1 cells/extracts sampled at the indicated time points, following release from a double thymidine block. (C) FACS profiles of PI‐stained MiaPaca‐2 cells following the indicated treatments. (D) Mean data plotted for two independent FACS experiments

Immunocytochemical staining revealed varying cellular levels of UHRF1 (Figure 5A). To determine whether this reflected variable UHRF1 expression during the cell cycle, pancreatic cancer cells were subjected to a double thymidine block and UHRF1 protein levels were measured over time following release (Figure 5B). UHRF1 levels were high in cells in S‐phase (0 h), relatively low as cells exited S‐phase (2 and 4 h post‐release), peaked at 7 h as cells accumulated in G_2_–M, and dropped significantly at 9 h as cells exited mitosis. To determine whether the peak in UHRF1 at G_2_–M was functionally important, cells were labelled with propidium iodide and FACS analysis conducted after UHRF1 depletion. Evidence of a G_2_–M block was observed in MiaPaca‐2 (Figure 5), Panc‐1 and CFPac‐1 (see supplementary material, Figure S6) and Suit‐2 (see supplementary material, Figure S7) cells following depletion of UHRF1. Two cell lines, MiaPaca‐2 and Suit‐2, were selected for further analysis. A substantial increase in the proportion of cells in G_2_–M was observed in MiaPaca‐2 cells following knockdown of UHRF1 (Figure 5C, 5D). Nrf2 knockdown caused a similar cell cycle profile, albeit with a more pronounced G_2_–M block. This similarity may reflect the fact that both UHRF1 and Nrf2 knockdown result in similar losses in Nrf2 protein levels (Figure 4H). Keap1 depletion increased the proportion of cells in G_1_, suggesting faster progression through the cell cycle. Finally, combined UHRF1 and Keap1 depletion rescued the effects of UHRF1 depletion on G_2_–M. Similarly, UHRF1 and Nrf2 depletion each caused a similar cell profile in Suit‐2 cells (S‐phase and G_2_–M block; see supplementary material, Figure S7), which was abrogated by the simultaneous depletion of UHRF1 and Keap1. These data suggest that suppression of Keap1 by UHRF1 contributes to the effective progression of PDAC cells through the cell cycle.

### UHRF1 expression in pancreatic tumours is associated with low Keap1 levels

**Figure 6:**
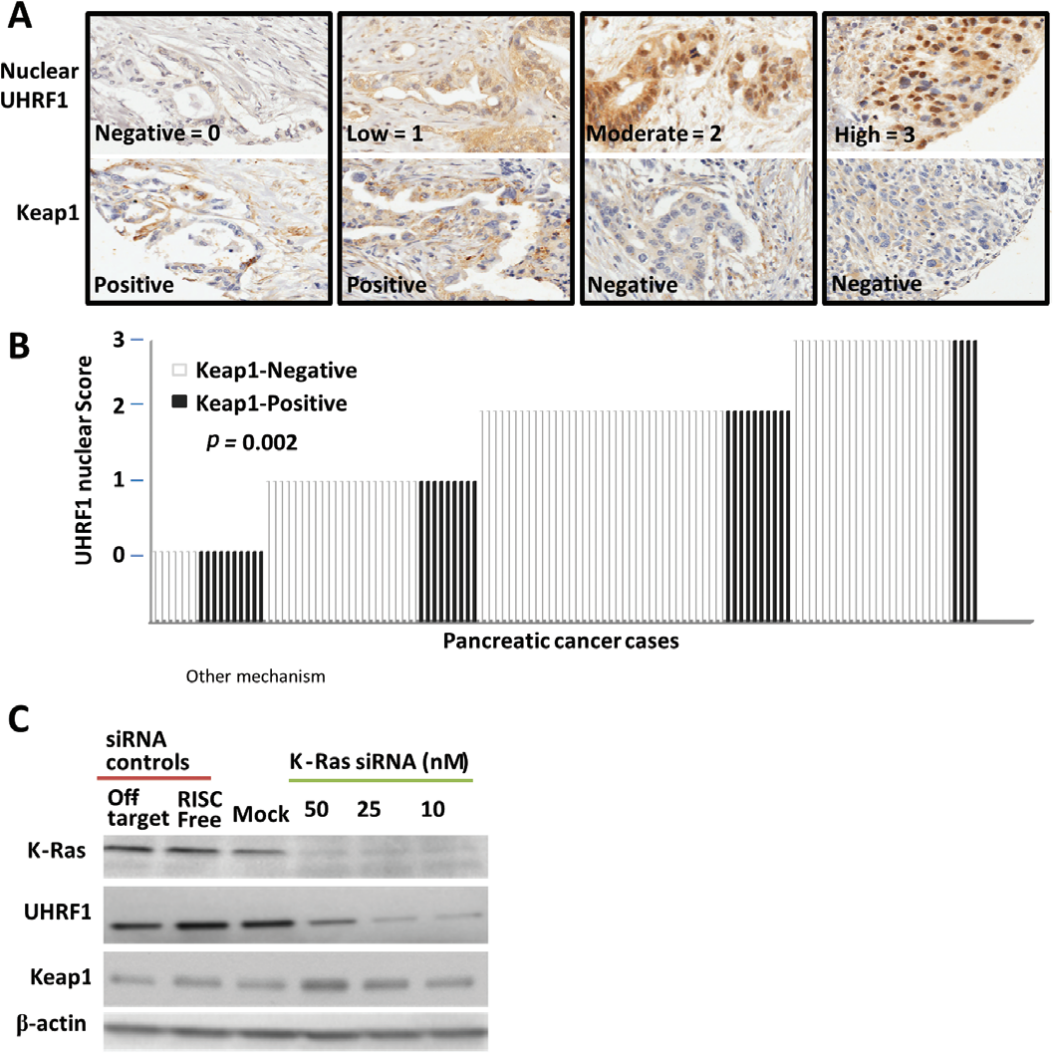
UHRF1 and Keap1 levels are inversely correlated in human PDAC tissues. (A) Immunohistochemistry of matched pancreatic cancer tissues stained for UHRF1 and Keap1. (B) The scores attributed for nuclear UHRF1 expression are plotted, grouped according to score and keap1 status; white bar, negative; black bar, positive. (C) Western blotting following K‐Ras knockdown

Based on our observed inverse relationship between UHRF1 and Keap1 levels in cell lines, we sought to determine whether this was relevant in patient tumour samples. Keap1 was evaluable by IHC (Figure 6A) for 124 patients. Of these, UHRF1 scores were attributed as follows: 17 scored 0 (negative); 32 scored 1 (weak); 46 scored 2 (moderate); and 29 scored 3 (strong). For patient samples with the highest UHRF1 levels (score 3), the majority (86%; 25/29) were negative for Keap1 expression (Figure 6B). In contrast, the majority (10/17) of patient samples lacking UHRF1 expression expressed Keap1. The percentages lacking Keap1 expression in the weak and moderate UHRF1 expressers were 72% and 78%, respectively (Figure 6B). Thus, the inverse association between UHRF1 and Keap1 levels observed in established human PDAC cell lines and primary mouse PDAC cells was upheld in PDAC tissue (Fisher's exact test, *p =* 0.002).

Finally, our observation that UHRF1 depletion had no effect on K‐Ras protein levels suggested that UHRF1 acts downstream of K‐Ras. To test this, we depleted K‐Ras from pancreatic cancer cells and probed for UHRF1 and Keap1. K‐Ras depletion was accompanied by diminished levels of UHRF1 and enhanced levels of Keap1 (Figure 6C), suggesting that active K‐Ras promotes expression of UHRF1.

## Discussion

Nrf2 protects cells from oxidants and electrophiles by stimulating the expression of genes involved in cytoprotection and detoxification 8. The cytoprotective role of Nrf2 is particularly important in cancers, including pancreatic cancer, where high levels of this transcription factor promote malignant cell proliferation and resistance to chemo‐ and radiotherapy 7. Oncogenic stimuli, including K‐Ras, can drive expression of Nrf2 in cancer cells 6. Here we describe an entirely novel mechanism regulating Nrf2 levels in pancreatic cancer cells. UHRF1 is an established epigenetic regulator contributing to maintenance of DNA methylation 37. We show that this function is maintained in PDAC cells and demonstrate for the first time that the *KEAP1* promoter is methylated in PDAC cell lines. We demonstrated that maintenance of *KEAP1* promoter methylation by UHRF1 may contribute to Nrf2 pathway regulation in Suit‐2 cells. However, this relationship was less definitive in MiaPaca‐2 and CFPac‐1 cells, suggesting that UHRF1 may control Keap1 protein levels through mechanisms other than *KEAP1* promoter methylation.

The inverse relationship between UHRF1 and Keap1 levels in human PDAC tissue samples substantiates the notion that UHRF1 contributes to the control of Keap1 levels *in vivo*. Most PDACs showed elevated nuclear UHRF1. In contrast, UHRF1 was observed in only a minority of benign epithelial ducts, with stronger expression observed in PanIN, suggesting that UHRF1 levels increase during the process of tumourigenesis. UHRF1 may contribute to PDAC growth. Depletion of UHRF1 was accompanied by an accumulation of cells in G_2_–M, as described previously for other cancer cell lines 38. Interestingly, *Nrf‐2*‐null cells have also been reported to arrest in G_2_–M 39. The presence of UHRF1 is associated with larger tumour size, although a link between UHRF1 expression and outcome was not established. Cui *et al* 40 reported an association between high UHRF1 expression and poor outcome in PDAC. The discrepancy in findings may be due to differences in scoring methods. We categorized patients as either negative for UHRF1 (score 0) or positive (score of 1, 2 or 3). Cui *et al* categorized patients as having low or high UHRF1 levels, and in our study a trend for poor outcome in high UHRF1 expressers (*p =* 0.07) was observed when patients are dichotomized into low (score 0 or 1) and high (score 2 or 3) expressers.

It is clear that the ability of UHRF1 to regulate Nrf2 levels contributes to the growth potential of pancreatic cancer cells. Depletion of UHRF1 led to a visible loss in cell number, as observed in non‐small cell lung cancer 25, colorectal cancer 23 and prostate cancer 26. Moreover, cells depleted of either UHRF1 or Nrf2 showed similar cell cycle profiles, supporting the concept that UHRF1 promotes tumour growth through up‐regulation of Nrf2.

The driver of UHRF1 expression in PDAC is likely to be oncogenic Ras. In our experiments, depletion of K‐Ras reduced UHRF1 levels, consistent with a recent report of enhanced UHRF1 expression in response to oncogenic Ras in pancreatic cancer cells 40. Together with our data, this supports a model whereby oncogenic Ras drives UHFR1 expression, which, in turn, maintains low Keap1 protein expression through *KEAP1* promoter methylation and/or other mechanisms. This allows Nrf2 levels to remain elevated and corresponding Nrf2 downstream proteins to be expressed, which then afford cellular protection from potential chemical and oxidative insults and promote growth. This proposed model of Nrf2 induction in pancreatic cancer is likely to work alongside other controlling forces, such as K‐Ras‐mediated transcriptional up‐regulation of Nrf2 6. Nrf2 activity is also regulated by the glycogen synthase kinase‐3 (GSK‐3)/β–transducin repeat‐containing protein (β‐TrCP) axis 41. Several signalling pathways regulate GSK‐3–β‐TrCP, including phosphatidyl inositol 3‐kinase (PI3K)–ATK 42, which lies downstream of K‐Ras. UHRF1 had no effect on *Nrf2* mRNA levels in our experimental systems, suggesting that it regulates Nrf2 through its effects on Keap1. However, UHRF1 levels alone were not sufficient to explain all Keap1 expression patterns in human pancreatic cancers, with occasional high UHRF1 expressers nonetheless expressing detectable Keap1, while some patients lacking UHRF1 also lacked Keap1. Thus additional control mechanisms are clearly in place governing the Keap1–Nrf2 system in PDAC.

In summary, we have uncovered a novel mechanism controlling the Keap1–Nrf2 cellular stress pathway, viz. UHRF1 suppression of Keap1 protein levels. This finding is of key importance to the range of cancers where over‐expressed UHRF1 and Nrf2 have been described, and may help explain their aggressive behaviour, including evasion of chemotherapy‐induced cytotoxicity.

## Author contributions

WA, TG and TN performed IHC; WA and TG undertook cell cycle analysis and luciferase, glutathione and ROS assays; WA performed methylation analysis under the supervision of TL; WA, TG and AO performed mRNA measurements and oxidative stress analysis; MGS generated KPC cells; WA, EC, JPN, TL, WG, LNB, CG, NK and BKP contributed to the experimental design; FC and TA reviewed pathology; RF performed K‐Ras knockdown; EC conceived the study and coordinated the work; EC, WA and JRS wrote the manuscript; and TL, FC, WG, LNB and CG reviewed and edited the manuscript.

**Supplementary material on the internet**

The following supplementary material may be found in the online version of this article:

Supplementary materials and methods

**Figure S1.** Primer sequences for promoter regions

**Figure S2.** DNA methylation levels of individual cytosines in the *RASSF1* promoter following UHRF1 depletion

**Figure S3.** DNA methylation levels of individual cytosines in the *RASSF1* and *CDKN2A* promoters following UHRF1 depletion

**Figure S4.** Additional data on the effects of UHRF1 and Nrf2 *in vitro*

**Figure S6.** FACS analysis of pancreatic cancer cells 72 h post transfection with control‐ or UHRF1‐targeting siRNA

**Figure S7.** UHRF1 contributes to progression through the cell cycle

## Supporting information


**AppendixS1.** Supplementary materials and methods

**Figure S1.** Primer designs for the targeted sequences in the promoter regions of LINE1, RASSF1, CDKN2A, KEAP1a and KEAP1b, generated using primer assay design (Qiagen). The grey‐blue vertical lines across the sequence indicates the location of each cytosine

**Figure S2.** Representative examples of DNA methylation levels of individual cytosines of the RASSF1 promoter in indicated cells following UHRF1 depletion

**Figure S3.** Representative examples of DNA methylation levels of individual cytosines of (A) the RASSF1 promoter and (B) the CDKN2 in indicated cells following UHRF1 depletion

**Figure S4.** Additional data on the effects of UHRF1 and Nrf2 in vitro. (A) Western blot analysis (of Suit‐2 extracts) harvested 24, 48 and 72 h following UHRF1 depletion, showing gain in Keap1 protein. (B) Western blot analysis (of CFPac‐1 extracts) harvested 72 h after UHRF1 depletion, showing gain in Keap1 protein and down‐regulation of HO‐1 and GcLc. (C) Relative luciferase activity following UHRF1 or Nrf2 depletion (MiaPaca‐2 cells). (D) Reactive oxygen species levels following the indicated treatments (Suit‐2 cells); **p < 0.01

**Figure S5.** UHRF1 contributes to pancreatic cancer cell growth. (A) Light microscopy image of MiaPaca‐2 cells following the indicated treatments. (B) Immunocytochemistry (ICC) for UHRF1 expression in PDAC cells transfected with control‐ or UHRF1‐targeting siRNA. (C) MTS analysis following UHRF1 and Keap1 knockdown (n = 3) in MiaPaca‐2 cells; *p < 0.01

**Figure S6.** FACS analysis of pancreatic cancer cells 72 h post‐transfection with control‐ or UHRF1‐targeting siRNA. An increase in cell accumulation in G_2_–M was observed, although the effect was modest in CFPac‐1 cells. Data are representative of three independent experiments

**Figure S7.** UHRF1 contributes to progression through the cell cycle: (A) histogram of PI‐stained Suit‐2 cells harvested 72 h after the indicated treatments; (B) with the mean data plotted for three independent experiments
