# Supplementary material for: UHRF1 regulation of the Keap1–Nrf2 pathway in pancreatic cancer contributes to oncogenesis
Source: J Pathol. 2015 Nov 30;238(3):423–33. doi: 10.1002/path.4665 (PMC4738372; doi:10.1002/path.4665)
Supplement: Supplementary file 1 — AppendixS1. Supplementary materials and methods [file PATH-238-423-s001.doc]

+A: **Supplementary materials and methods**

+B: Small Interference RNA (siRNA)-mediated knockdown of UHRF1 and Keap1

All siRNAs were from GE Healthcare ([Little Chalfont](http://en.wikipedia.org/wiki/Little_Chalfont), [Buckinghamshire](http://en.wikipedia.org/wiki/Buckinghamshire), [UK](http://en.wikipedia.org/wiki/United_Kingdom)). UHRF1 depletion was performed using Human UHRF1 targeting siRNAs 1–4 (siRNA 1, GCCAUACCCUCUUCGACUA; siRNA 2, GGAACAGUCUUGUGAUCAG; siRNA 3, UGGAGGAGGACGUCAUUUA; and siRNA 4, GAACGGCGUGGUCCAGAUG), human Keap1 targeting siRNAs 2 and 4 (siRNA 2, CAGCAGAACUGUACCUGUU; and siRNA 4, CGAAUGAUCACAGCAAUGA), human Nrf2 targeting siRNA (D-003755-05) and mouse UHRF1 targeting siRNA (GAAACGCGGCUUCUGGUAU). Controls included non-targeting siRNA control 1 and RISC-free siRNA. Typically, 2 × 10^5^ cells were seeded in six-well plates (Corning BV Life Sciences, Amsterdam, The Netherlands) and were transfected at 40% confluence with 30 nm siRNA, using Lipofectamine 2000 (Life Technologies) in antibiotic-free medium, according to the manufacturer’s instructions, and harvested at 72 h or the indicated times.

+B: Western blotting

The primary antibodies used in this study were: anti-UHRF1 (1:1000; ab57083, Abcam, Cambridge, UK); anti-UHRF1 (1:1000; sc-136264, Santa Cruz Biotechnology); anti-Keap1 (1:1000; sc-15246, Santa Cruz Biotechnology); anti-Nrf2 (1:1000; ab137550, Abcam); anti-P16 (1:500; sc373695, Santa Cruz Biotechnology); anti-caspase 3 (1:1000; ab13847, Abcam); anti-Cyclin A (1:2000; sc751 Santa Cruz Biotechnology); anti-Cyclin D (1:2000; ab137875, Abcam); anti-Cyclin E (1:2000; sc248, Santa Cruz Biotechnology); anti-HO-1; anti-GcLc (1:3000; Santa Cruz Biotechnology); anti-β-actin (1: 20 000); anti-CYP2A5, mouse homologue of CYP2A6, was from Risto Juvonen, University of Eastern Finland, Kuopio, Finland (1:3000); and anti-K-Ras (1:500; ab55391, Abcam). Anti-mouse antibodies were from Santa Cruz Biotechnology, including: anti-UHRF1 (1:1000; M-132); sc-98817; anti-Keap1 (1:1000; H-190); sc-33569; anti-Nrf2 antibody (1:1000; C-20); and sc-722. Secondary antibodies were: goat anti-mouse (Dako, P0447), goat anti-rabbit (Dako, P0448) and rabbit anti-goat (Dako, P0449) (all HRP-conjugated; 1:3000; Dako, Ely, UK); and rabbit anti-chicken (HRP-conjugated, 1:5000; Sigma, A9046).

+B: RNA extraction and quantitative real-time PCR (qRT–PCR)

The following primers were designed using Primer Express Software (Applied Biosystems, UK). *KEAP1*, forward 5′-CAG ATT GGC TGT GTG GAG TT-3′, reverse 5′-GCT GTT CGC AGT CGT ACT TG-3′; *GAPDH*, forward 5′-GGC CTC CAA GGA GTA AGA CC-3′, reverse 5′-AGG GGT CTA CAT GGC AAC TG-3′.
