# Supplementary figures and images for: UHRF1 regulation of the Keap1–Nrf2 pathway in pancreatic cancer contributes to oncogenesis
Source: J Pathol. 2015 Nov 30;238(3):423–33. doi: 10.1002/path.4665 (PMC4738372; doi:10.1002/path.4665)

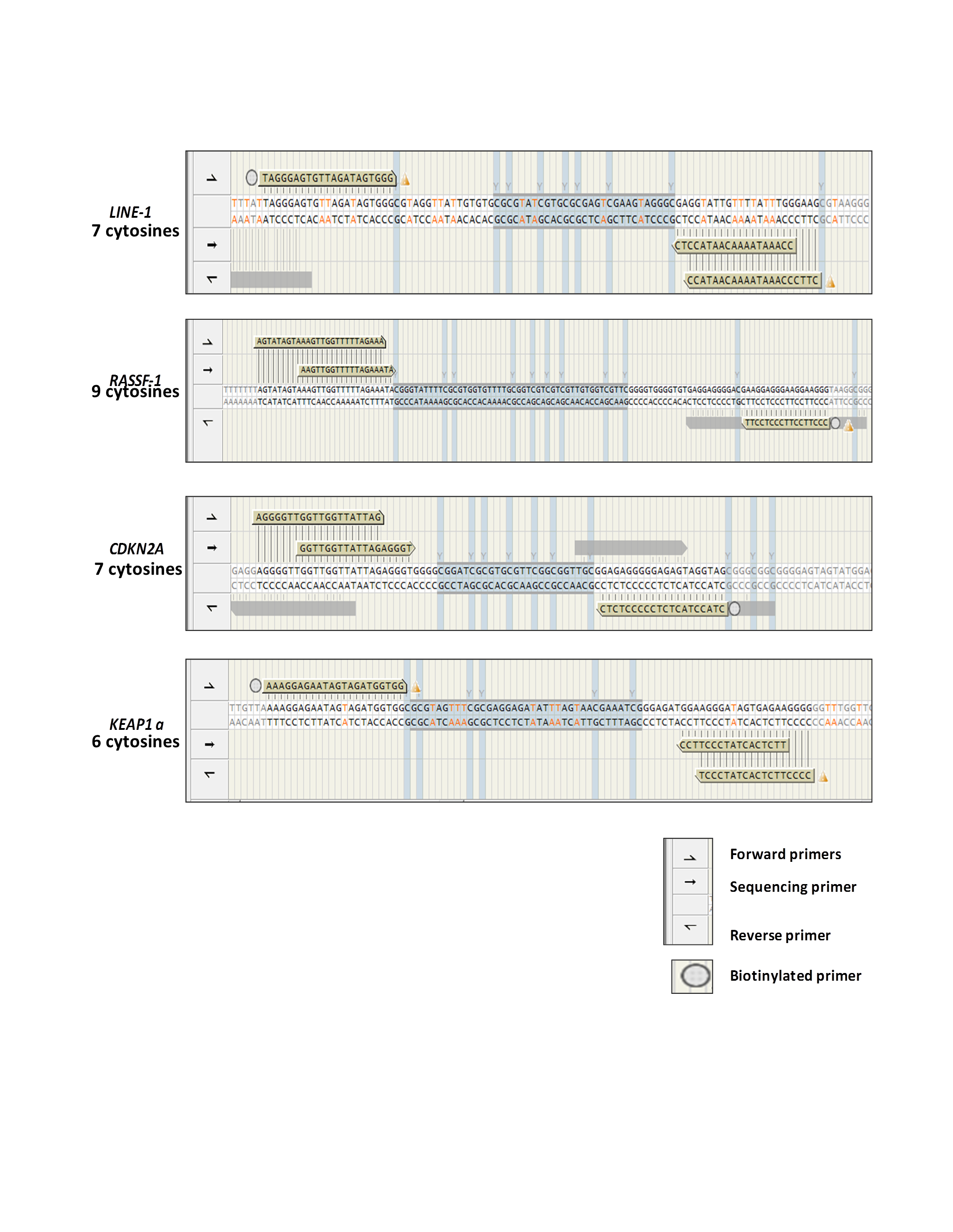

Supplement: Supplementary file 2 — Figure S1. Primer designs for the targeted sequences in the promoter regions of LINE1, RASSF1, CDKN2A, KEAP1a and KEAP1b, generated using primer assay design (Qiagen). The grey‐blue vertical lines across the sequence indicates the location of each cytosine [file PATH-238-423-s002.tif]

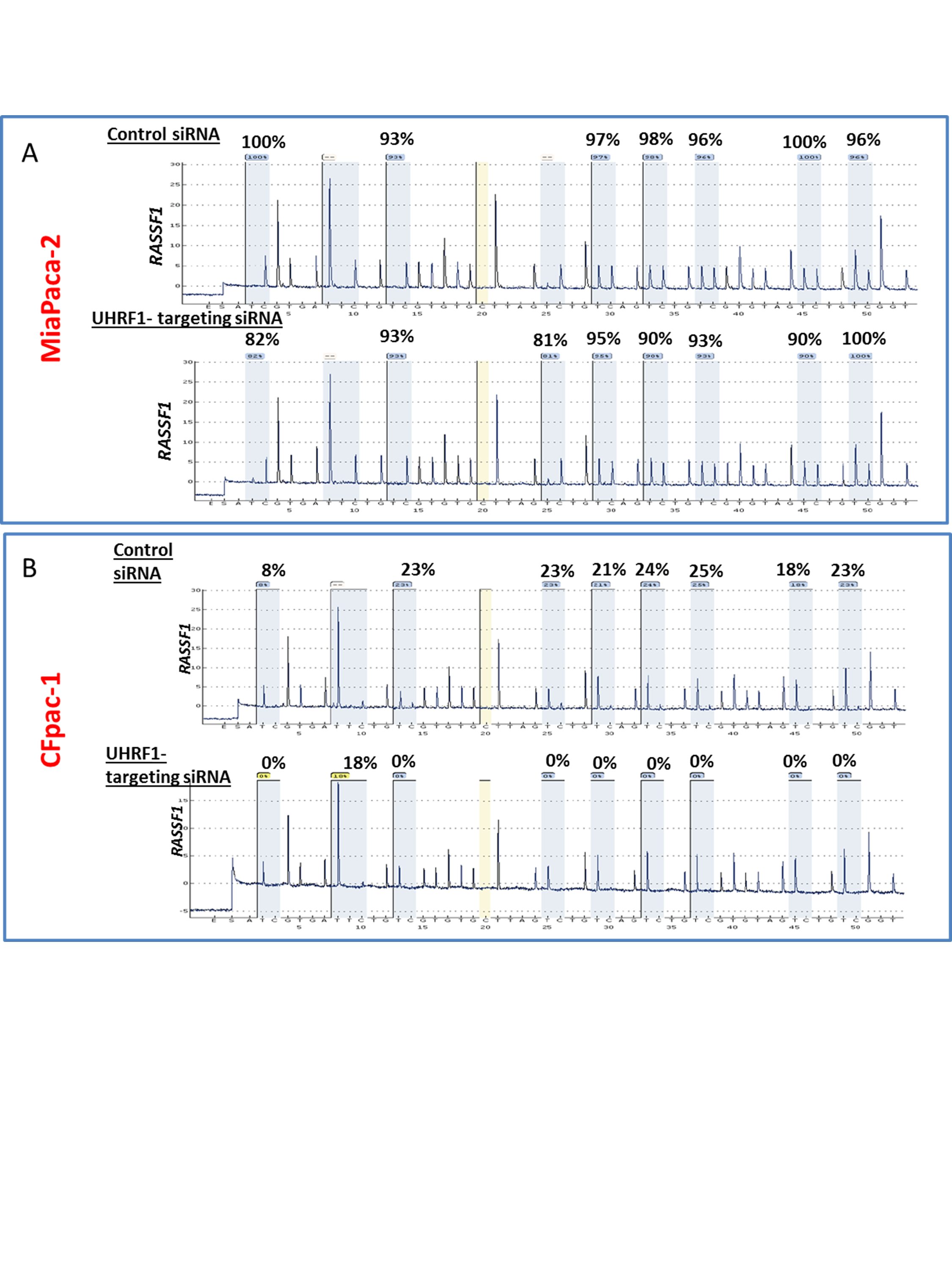

Supplement: Supplementary file 3 — Figure S2. Representative examples of DNA methylation levels of individual cytosines of the RASSF1 promoter in indicated cells following UHRF1 depletion [file PATH-238-423-s003.tif]

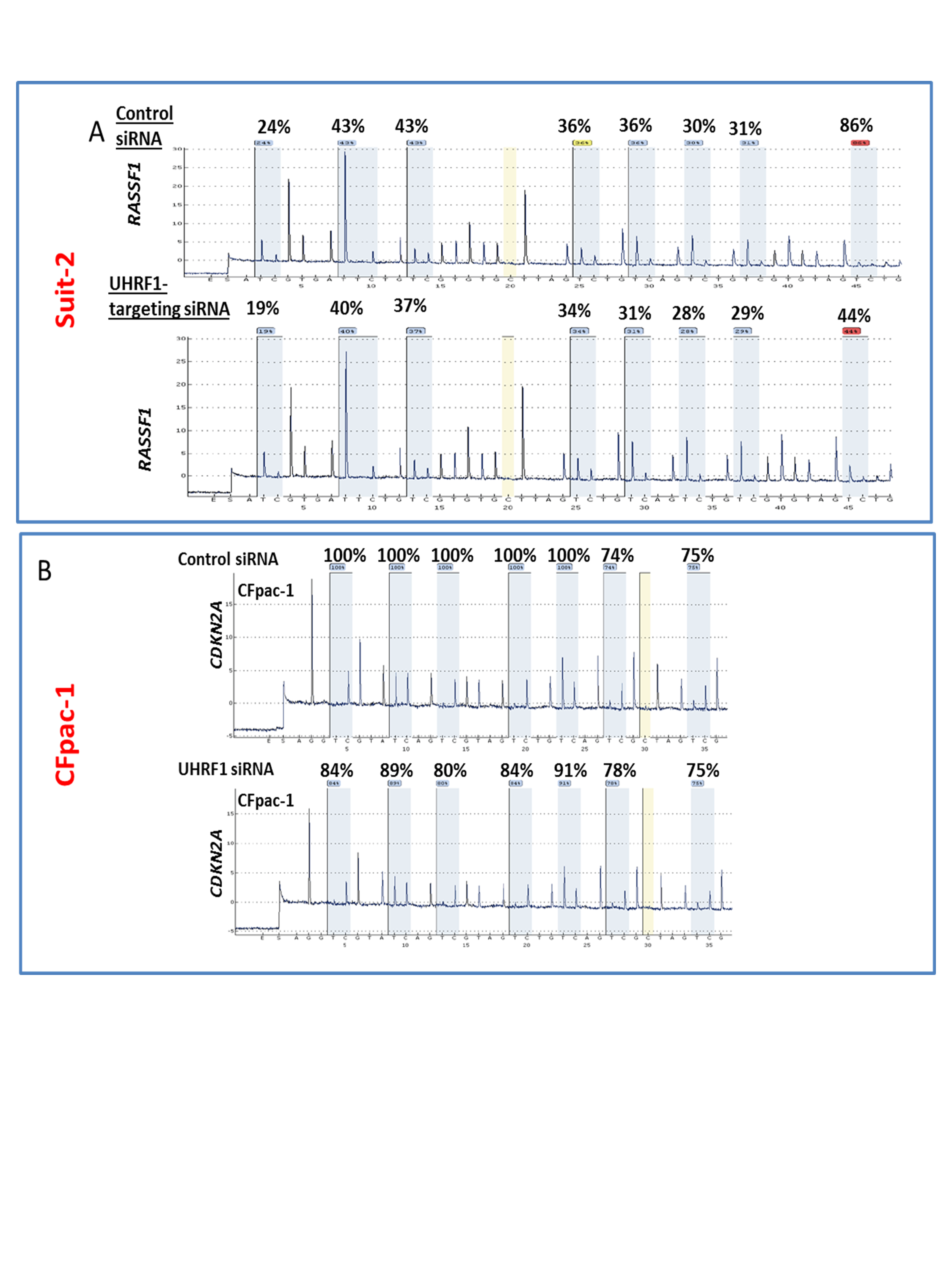

Supplement: Supplementary file 4 — Figure S3. Representative examples of DNA methylation levels of individual cytosines of (A) the RASSF1 promoter and (B) the CDKN2 in indicated cells following UHRF1 depletion [file PATH-238-423-s004.tif]

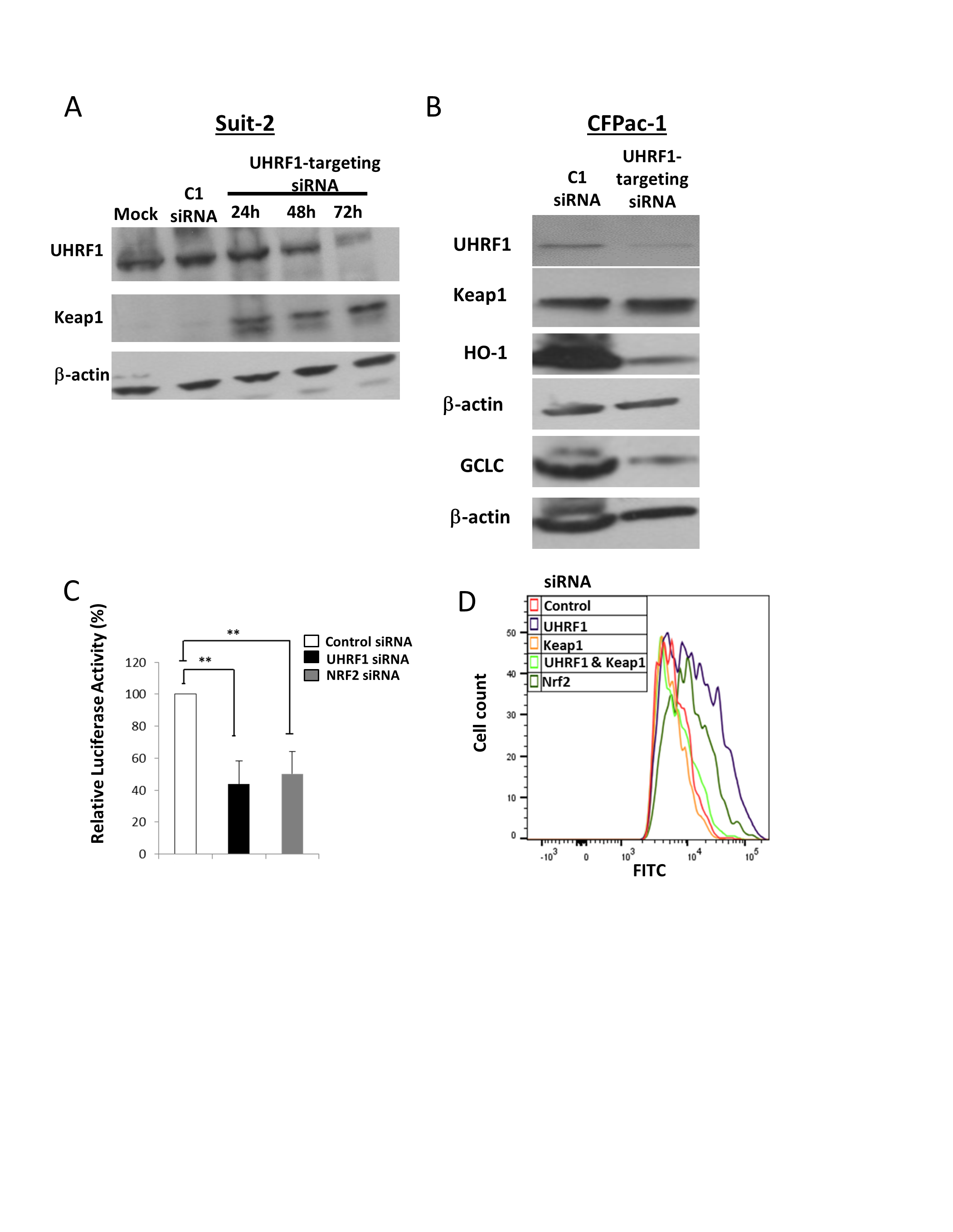

Supplement: Supplementary file 5 — Figure S4. Additional data on the effects of UHRF1 and Nrf2 in vitro. (A) Western blot analysis (of Suit‐2 extracts) harvested 24, 48 and 72 h following UHRF1 depletion, showing gain in Keap1 protein. (B) Western blot analysis (of CFPac‐1 extracts) harvested 72 h after UHRF1 depletion, showing gain in Keap1 protein and down‐regulation of HO‐1 and GcLc. (C) Relative luciferase activity following UHRF1 or Nrf2 depletion (MiaPaca‐2 cells). (D) Reactive oxygen species levels following the indicated treatments (Suit‐2 cells); **p < 0.01 [file PATH-238-423-s005.tif]

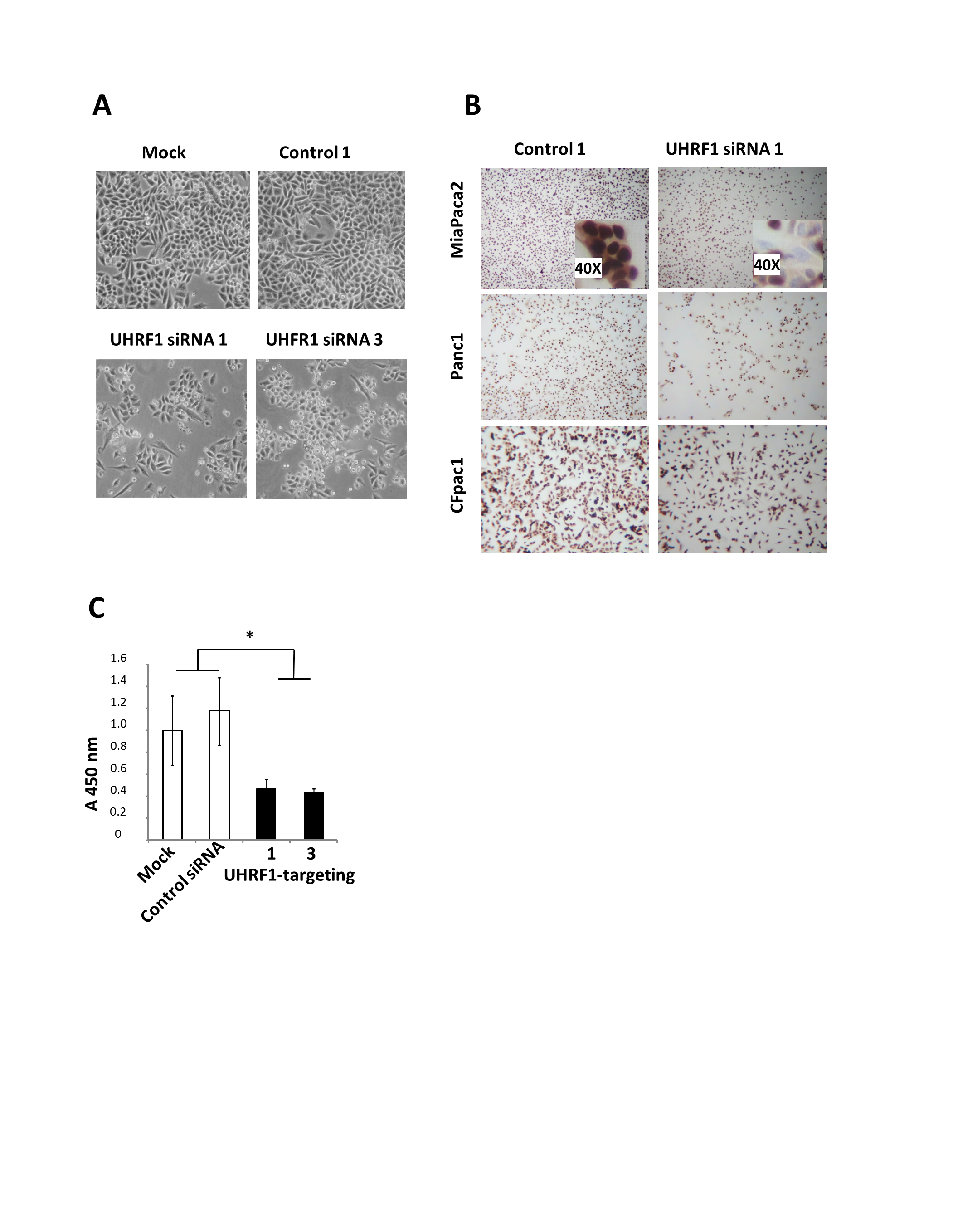

Supplement: Supplementary file 6 — Figure S5. UHRF1 contributes to pancreatic cancer cell growth. (A) Light microscopy image of MiaPaca‐2 cells following the indicated treatments. (B) Immunocytochemistry (ICC) for UHRF1 expression in PDAC cells transfected with control‐ or UHRF1‐targeting siRNA. (C) MTS analysis following UHRF1 and Keap1 knockdown (n = 3) in MiaPaca‐2 cells; *p < 0.01 [file PATH-238-423-s006.tif]

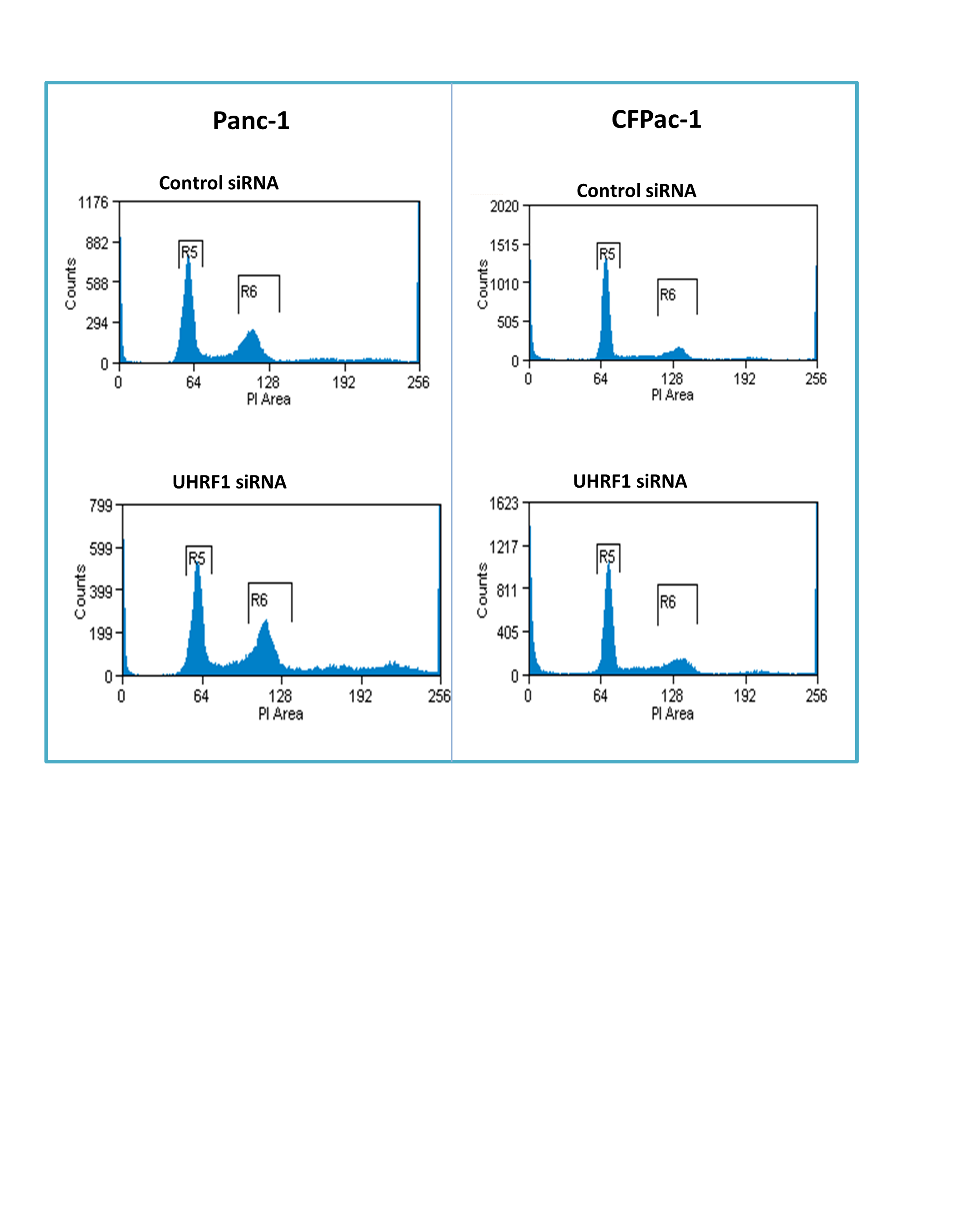

Supplement: Supplementary file 7 — Figure S6. FACS analysis of pancreatic cancer cells 72 h post‐transfection with control‐ or UHRF1‐targeting siRNA. An increase in cell accumulation in G2–M was observed, although the effect was modest in CFPac‐1 cells. Data are representative of three independent experiments [file PATH-238-423-s007.tif]

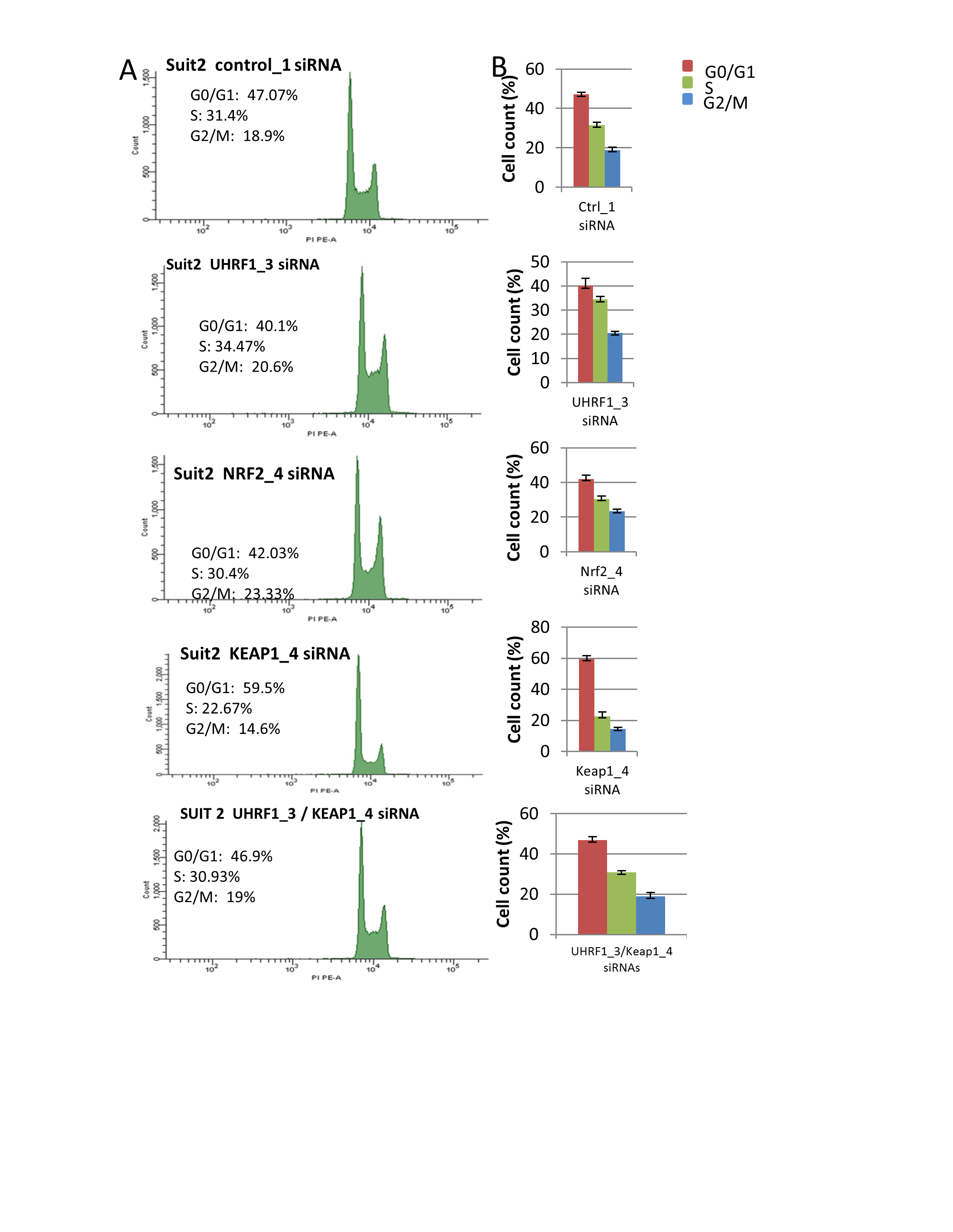

Supplement: Supplementary file 8 — Figure S7. UHRF1 contributes to progression through the cell cycle: (A) histogram of PI‐stained Suit‐2 cells harvested 72 h after the indicated treatments; (B) with the mean data plotted for three independent experiments [file PATH-238-423-s008.tif]
